# Plasma-induced modification of fenugreek galactomannan thickener film via dielectric barrier discharge for textile printing

**DOI:** 10.1038/s41598-025-24053-0

**Published:** 2025-11-10

**Authors:** Hend M. Ahmed, J. I. Abd–El Thalouth, E. Khaled, U. M. Rashed, A. A. El-Halwagy, Tawfik A. Khattab

**Affiliations:** 1 Dyeing, Printing and Auxiliaries Department, Textile Research and Technology Institute, National Research Centre, Cairo, 12622 Egypt; 2 Textile Printing, Dyeing and Finishing Department, Faculty of Applied Art, Helwan University, Cairo, Egypt; 3 Physics Department, Faculty of Science, Al-Azhar University, Cairo, Egypt

**Keywords:** Fenugreek (*Trigonella foenum graecum* l.)-based polysaccharide, Surface modification, Plasma-supported extraction, Plant sciences, Environmental sciences, Chemistry, Engineering, Materials science

## Abstract

Fenugreek seeds are a valuable source of galactomannan, a natural thickener with applications in textile printing. However, traditional galactomannan extraction methods often suffer from lengthy processing times and limited yields. To address these limitations, the use of dielectric barrier discharge (DBD) atmospheric cold plasma was applied as a pretreatment method. Dried fenugreek seeds were subjected to air and oxygen gas plasma treatments at various discharge powers and exposure durations. Subsequently, galactomannan was extracted and formed into films (membranes) for characterization. Additionally, the rheological properties of the galactomannan solutions were investigated to assess their potential as eco-friendly thickening agents in textile printing pastes. The antibacterial activity of the treated galactomannan was also investigated.

## Introduction

Printing is the most significant process used to improve the aesthetic appeal of textile materials. Thickeners are used to restrict the coloring matter on a certain design during the printing process^1–3^. They are a viscous matrix that can impart stickiness and plasticity to the printing paste, facilitating the dyeing process on the fabric surface without bleeding. The thickening agents have shown various functions in textile printing, including providing viscosity to the printing paste, holding the ingredients of the print paste on the fabric surface, and preventing premature reaction between the chemical agents enclosed in the print paste^4,5^. In order to identify a material as a good thickener, it is necessary to take into account various requirements, including minimum effect on the color yield, ease of removal, good adhesion to the fabric surface, print paste stability, appropriate viscosity, and low cost^6–8^. However, most thickeners are synthetic materials that are harmful to the environment and human health. Thus, protecting the environment has been a major global concern, particularly in the textile and clothing industry. Thus, the use of environmentally friendly materials, such as natural thickeners, has been a challenge. The sources of natural thickeners have been reported from various plant sources. The constituents of a natural thickener must be nonallergic and nontoxic to humans. A good thickener for textile printing must be soluble in water and absorb water to form a viscous solution^9–12^.

Fenugreek (*Trigonella foenum graecum* L.) is an excellent resource of galactomannan; however, the traditional techniques used to extract galactomannan are commonly time-consuming and costly due to the relatively low recovery rate^13^. Fenugreek gums were firstly used in industry as a source of galactomannan, a polysaccharide of galactose combined with manna, a high molecular compound of mannose. The ratio of galactose to mannose in fenugreek was reported as 1:1 as compared to guar and locust bean gums that have the ratio of 1:2 and 1:4 respectively^14^. However, natural thickeners have shown major drawback as they get rotten after a certain period of time and become unsuitable for printing pastes^15,16^. To overcome this drawback and the extent of the thickener period validity, we propose to expose it to plasma treatment. Plasma technology is an attractive future-oriented technique due to its environmental suitability and varied applications. It has already been employed to induce surface modification of textile material, leading to enhanced textile properties such as wettability, liquid repellency, coating adhesion, and dyeability^17–19^. The depth of a plasma-induced surface modification ranges from 100 to several micrometers while retaining the mechanical performance, bulk properties, and physiochemical performance of the original material^20,21^. Atmospheric plasma has been successfully employed to change the surface functionalities, where it transfers hydrophobic surfaces to hydrophilic by generating polar substituents on the fabric surface^22,23^. It can also modify the physicochemical features of the fabric surface^24^. Cold atmospheric plasma has been generated in a dielectric barrier device (DBD) to improve the surface characteristics of textiles^25^. DBD plasma has been utilized to amend both structure and function of lipids, starches, and proteins to improve their rheological properties^26,27^.

Plasma treatment has demonstrated a remarkable ability to transform guar gum by reducing its molecular weight, enhancing its emulsion capacity, and altering its rheological properties while also increasing surface porosity. Similar enhancements have been observed in gum Arabic and xanthan, where modifications effectively boosted their viscosity and elastic behavior. Yet, a significant research gap remains: locust bean gum, another widely used galactomannan, has yet to be explored under plasma treatment, and no comparative studies featuring consistent treatment protocols have been published^28,29^.

In this context, limited studies have been reported on the impact of DBD plasma on non-starchy sugars, such as galactomannan. Furthermore, while dielectric barrier discharge (DBD) plasma is recognized for its ability to modify crop surfaces—enhancing permeability, decontamination, and surface activation^30^—its application in processing natural extracts, particularly non-starchy sugars like galactomannan, remains underexplored. This study investigates the impact of DBD plasma on the rheological behavior of galactomannan extracted from fenugreek seeds. We demonstrate that plasma treatment extended the usable storage period of the thickener from 5 to 15 days and endowed the resulting film with multifunctionality suitable for food, medicine, and polymer industries. The physicochemical properties of the extracted galactomannan films were studied using a range of techniques, including scanning electron microscopy (SEM), transmission electron microscopy (TEM), energy-dispersive X-ray analysis (EDX), and Fourier transform infrared (FTIR) spectroscopy. The functional groups and elemental composition were also explored. Furthermore, the plasma-treated thickener’s interaction with various fabrics and dyestuffs was evaluated under different plasma treatment conditions. The findings of this research demonstrate that DBD plasma technology offers a green alternative for enhancing the extraction efficiency of fenugreek galactomannan and modifying its functional properties. By reducing the reliance on harsh chemicals and improving extraction effectiveness, this approach facilitates more diverse applications of fenugreek galactomannan in the food, pharmaceutical, and polymer industries.

## Experimental

### Materials

#### Fenugreek seed

Dry Fenugreek (*Trigonella foenum graecum* L.) was obtained from Harraz for Food Industry & Natural Products Co. (Cairo, Egypt). The seeds were manually separated and stored under cool and dry conditions for further experiments. For illustration, a study investigated various drying methods for fenugreek greens, including hot air drying at 40 °C with relative humidity ranging from 58% to 63%. This temperature was found to be effective in preserving the color and nutritional quality of the greens.

#### Extraction of Galactomannan

In order to extract the gum of galactomannan from the seeds of the fenugreek plant^31^, the endosperms were isolated from the germ and the hull. The seeds were firstly rinsed with distilled water to get rid of impurities, air-dried, and then crushed mechanically. The provided powder was sieved to get rid of the germ that has the lowest hardness as compared to the other components. The hull and the endosperm, which consist mainly of the galactomannan gum, were immersed in water overnight to allow the swelling process. Using a fine silk fabric, the swelled gum was separated from the hull by filtration.

#### Cold plasma experimental setup

**Fig. 1:**
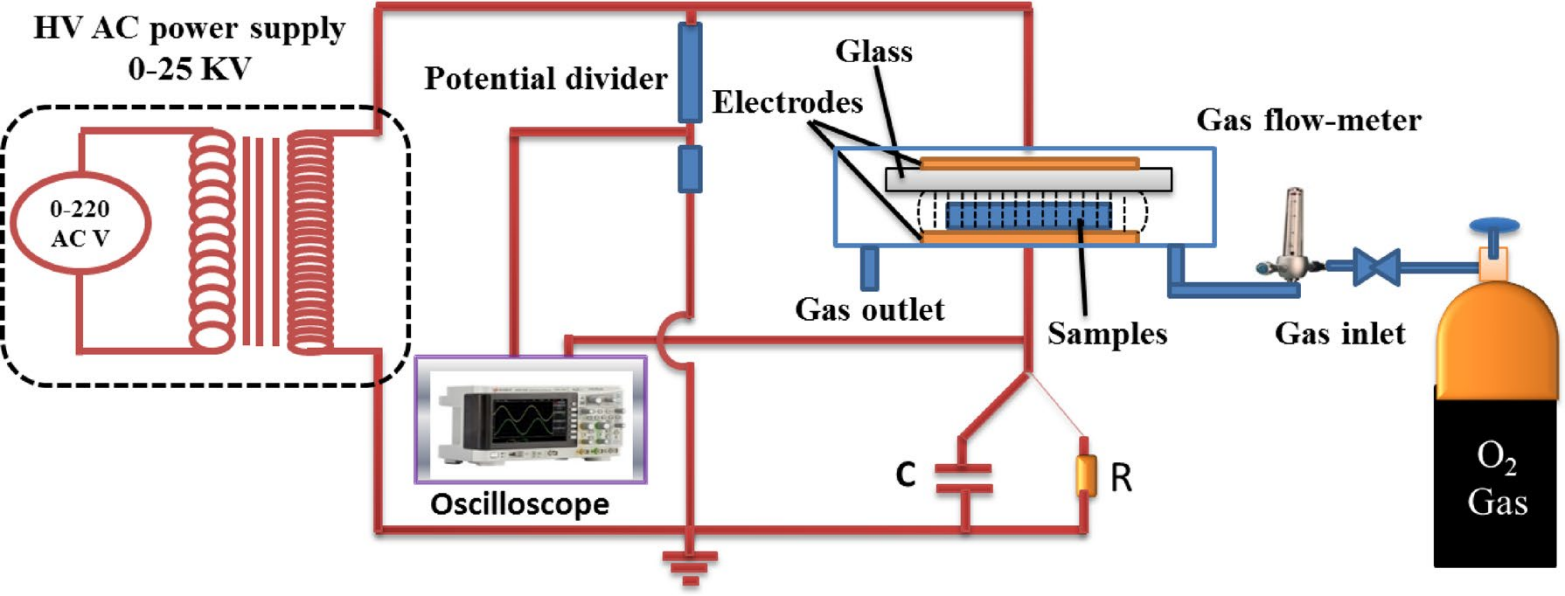
Diagram of cold atmospheric DBD plasma generator.

As represented in Fig. 1, DBD reactor is composed of two parallel electrodes. The lower electrode is a stainless-steel disc (25 cm^**2**^ × 25 cm^**2**^), which is resistant to oxidation and corrosion upon exposure to ozone. The upper electrode is an aluminum film (25 cm^**2**^ × 25 cm^**2**^) placed on a dielectric disc of glass (2 mm). The electrode edge was smoothened to prevent localization of the electric field at the edges of the electrodes. The distance between the lower electrode and the dielectric glass plate was 2 mm enclosed in a Perspex chamber with a gas inlet and outlet. Plasma discharges are generated by an AC power supply (50 Hz frequency; 25 kV/30 mA) attached to the upper electrode, whereas the lower electrode is attached to earth via a capacitor (C) of 3.35 mF or a resistance R of 100 Ω. The power supply is made up of a step-up transformer immersed in an oil and a potentiometer. The samples were positioned between the two electrodes. The gas was allowed to flow through a gas flow meter into the sample, where the electric discharge was produced as the DBD is operated under atmospheric pressure. The working gases that were used in the treatment process were air and oxygen. Plasma cleaning was applied under various gas flow rates and different treatment durations. The voltage was determined by a resistance potential probe (high-voltage probe), which is linked in parallel with the discharge electrodes. The current was determined by determining the voltage crosswise and the resistance across a digital storage of two-channel oscilloscope (GDs-1072-u GWinsTEX, 70MHZ). The power was determined by a capacitor to compute the charge flow in the cell.

#### Plasma treatment

**Fig. 2:**
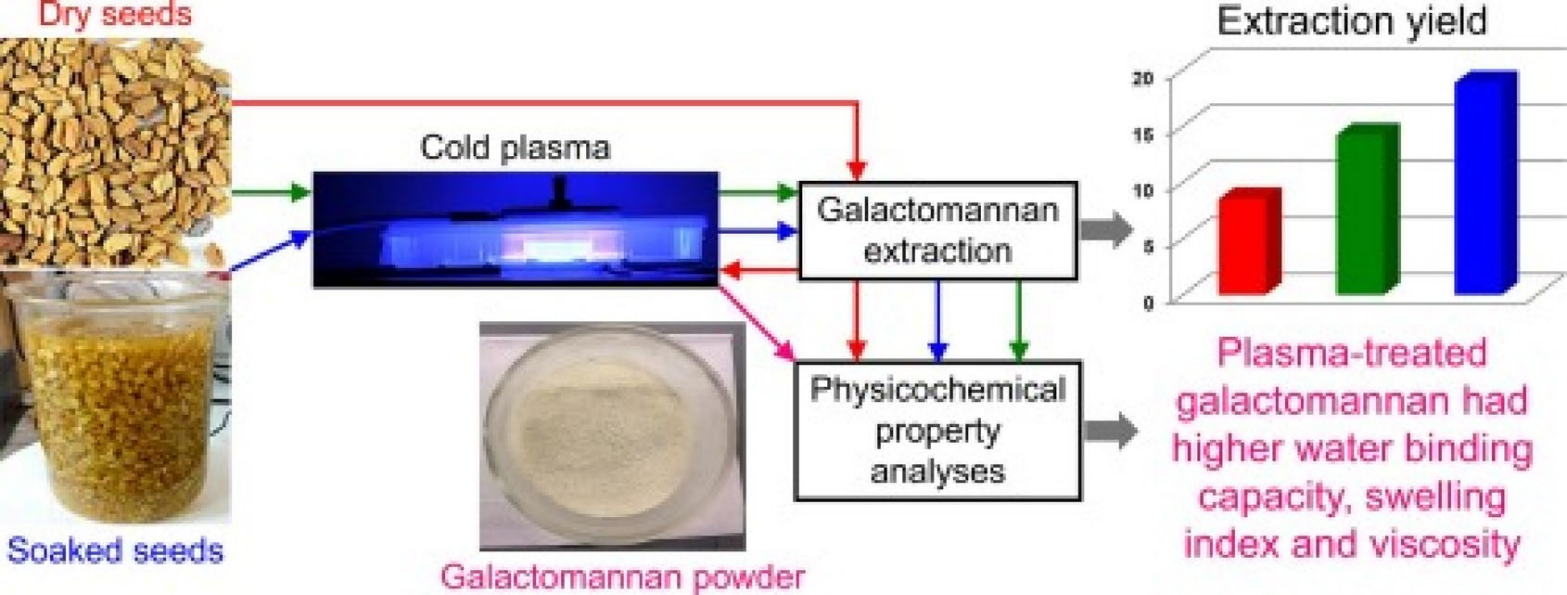
Cold plasma treatment of fenugreek galactomannan.

The plasma treatment of the prepared galactomannan film was adjusted according to the procedure shown in Fig. 2. Firstly, the fenugreek seeds were rinsed with distilled water to get rid of both impurities and sediment. The seeds were air-dried for a day and then milled with an industrial mixer to provide the seeds separated from the hard outer part. The seeds (250 g) were soaked in distilled water (1 L), and the provided paste was filtered through a piece of silk to produce a thickener. The stock was drop-cast in Petri dishes and dried in a digital oven at 105 °C for 30 min to generate a film. The provided film was exposed to both air and oxygen gas plasma, using discharge powers of 17.35 W and 12.2 W for oxygen plasma at a flow rate of 0.5 L/min and 15.9 W and 10.15 W for air plasma at different exposure durations, including 10, 20, and 30 min.

### Characterization and measurements

#### Rheological studies

The rheological features of the printing paste were evaluated by a Rheomat-12 viscometer (Zurich, Switzerland). Shear stress was measured as a function of shear rate, with readings taken after a shearing period of 60 s to ensure equilibrium. The viscosity (µ) of the paste was calculated by the following equation^32^:

$${\rm \mu=\tau/D}$$

where µ (poise) is the viscosity, D is the shearing rate, and τ is the shearing stress (dyne.cm^−2^).

#### Scanning electron microscopy (SEM)

Both plasma-treated and untreated samples were examined using a JSMT-20 scanning electron microscope (JEOL, Japan) at a resolution of 200 Å, a magnification range of 1500–2000×, and an accelerating voltage of 19 kV. Prior to imaging, the films were cut into approximately 2 cm × 2 cm pieces and thoroughly dried. The sample surface was then coated with a thin layer of gold using a sputter coater to enhance the sample conductivity and prevent surface charging. Gold coating, typically applied at a thickness of 5–20 nm, provides a fine-grained conductive layer that improves image contrast and signal quality, particularly in secondary electron (SE) mode. Nanoparticle diameters were measured using ImageJ software, following appropriate image calibration and thresholding procedures.

#### Energy-dispersive X-ray (EDX)

Elemental composition of both plasma-treated and untreated samples was analyzed using a TEAM/EDX system coupled with a JSMT-20 SEM (JEOL, Japan) at an accelerating voltage of 20 kV. The working distance was maintained at [insert WD, e.g., 10 mm], and the spot size was set to [insert spot size if known] to optimize spatial resolution. Data acquisition was conducted over a collection time of [insert time, e.g., 60 s] per analysis point. The EDX system was equipped with an [Si (Li) or Silicon Drift Detector (SDD)], with SDD being preferred for its improved energy resolution and faster processing capabilities. A thin conductive gold coating, approximately [insert thickness, e.g., 10 nm], was applied to all samples via sputter coating to prevent surface charging during imaging. While this enhances SEM imaging quality, it may obscure the detection of lighter elements such as carbon, oxygen, and nitrogen in EDX analysis due to the high atomic number of gold. Under the selected operating conditions, the X-ray penetration depth is estimated to be in the range of 1–2 μm, depending on the density and composition of the sample, meaning the analysis reflects subsurface as well as surface chemical information.

#### Transmission electron microscopy (TEM)

TEM (JEM-1230, JEOL, Japan) was employed to measure the particle diameter, size distribution, and morphology at an accelerating voltage of 120 kV. A drop of a colloid solution was deposited on a 400-mesh copper grid coated with an amorphous carbon layer, and then the solvent was air-dried for TEM analysis. TEM analysis was performed by a JEM-1230 (JEOL, Japan) operated at an accelerating voltage of 120 kV to evaluate the morphology, particle diameter, and size distribution. Prior to analysis, the colloid solution was diluted with deionized water to reduce aggregation and ensure uniform deposition. A drop of a diluted sample was poured onto a 400-mesh copper grid and then allowed to air-dry under ambient conditions before imaging. Image analysis was conducted using ImageJ, where particle diameters were measured from TEM micrographs after appropriate thresholding and calibration using the microscope’s scale bar. Size distribution histograms were generated based on measurements of [number, e.g., 100 or more] randomly selected particles to ensure statistical significance. The data were analyzed to calculate mean particle size and standard deviation.

#### Fourier-transform infrared spectrophotometer (FTIR)

FTIR spectra (400–4000 cm⁻¹) were recorded using a Nexus-670 spectrometer (Nicolet, United States) following a previously reported procedure. The samples were prepared using the ATR (attenuated total reflectance) method. Each spectrum was collected at a resolution of 4 cm⁻¹ scans were averaged to improve the signal-to-noise ratio. A background spectrum was measured and automatically subtracted from each sample spectrum to eliminate atmospheric interference and instrument noise.

#### Antibacterial activity

A stock culture of *Staphylococcus aureus* (100 µl with colony-forming units (CFU) value of 2.4 × 10^7^) was inoculated in a nutrient broth (10 mL) enclosing a peptone (5 g/L) and a beef extract (3 g/L) with a pH value of 6.8 in an Erlenmeyer flask (100 mL) and then incubated for 24 h. The films were inserted into the inoculated flask enclosing 20 µl of inoculum. After 16 h of incubation at 37 °C, both sample-free culture (control) and sample-containing culture were diluted in the range of 10^−1^−10^−5^. The CFU value was measured by inoculating Petri dishes enclosing agar media of a solidified nutrient with 100 µl of every dilution. The growth reduction rate (R) was determined using the following equation^33^:

$${\rm R (\%) = [(B-A)/B] \times\:100}$$

Where A is the CFU/mL for each sample-containing culture after 16 h of incubation, and B is the CFU/ml for the control after 16 h of incubation.

#### Statistical analysis

Employing Prism GraphPad software (version 6.0, San Diego, CA, United States), the findings were reported as an average ± standard deviation (SD). The One-way *ANOVA* was employed to determine the statistical significance as the p-value was calculated to approximate the difference amid groups.

## Results and discussion

### Plasma treatment

**Fig. 3:**
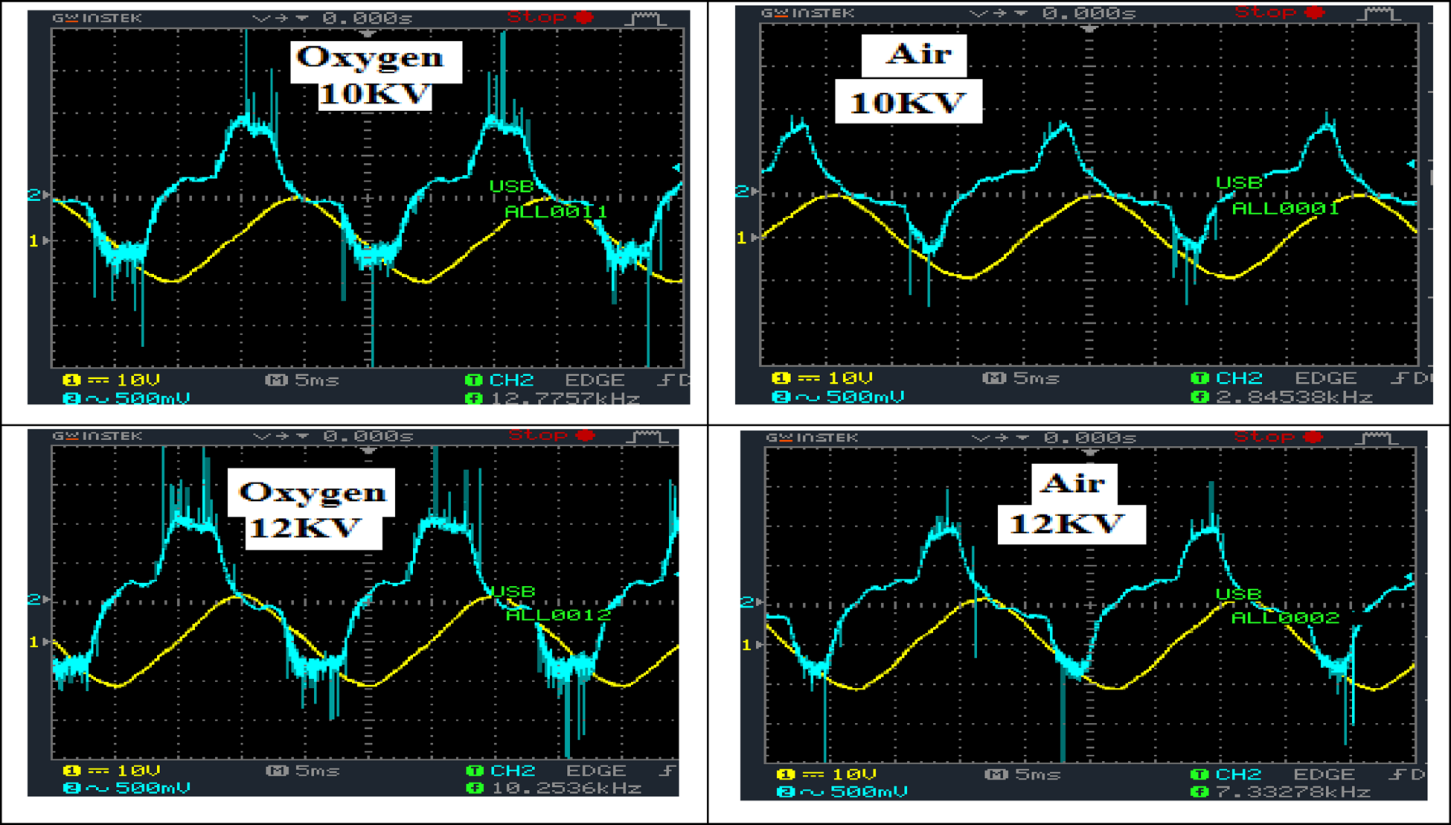
Waveforms of voltage and current of DBD for oxygen and air plasma at peak voltages of 10KV and 12 KV.

Figure 3 displays the voltage and current waveforms from the oscilloscope for oxygen and air plasma at peak voltages of 10 kV and 12 kV. A stream discharge was generated, which is distinguished by discrete current spikes correlated to the creation of micro-discharges (filaments) of tens of nanoseconds ^23–25^. Those micro-discharges spread randomly in the discharge region. They start upon locally reaching the breakdown field and are extinguished upon reducing the field to the value at which the electron recombination predominates over ionization. Owing to the buildup of a charge on the dielectric field surface, the micro-discharge collapses within a few nanoseconds after breakdown, ending the flow of current. When the external voltage was increased, more micro-discharges were begun at new locations due to decreasing the residual charges on the dielectric plate. Because the high voltage at the low frequency tends to distribute the micro-discharges and increase the number of instant filaments^24–26^, the spike peaks increase with raising the voltage peak.

### Power measurements

**Fig. 4:**
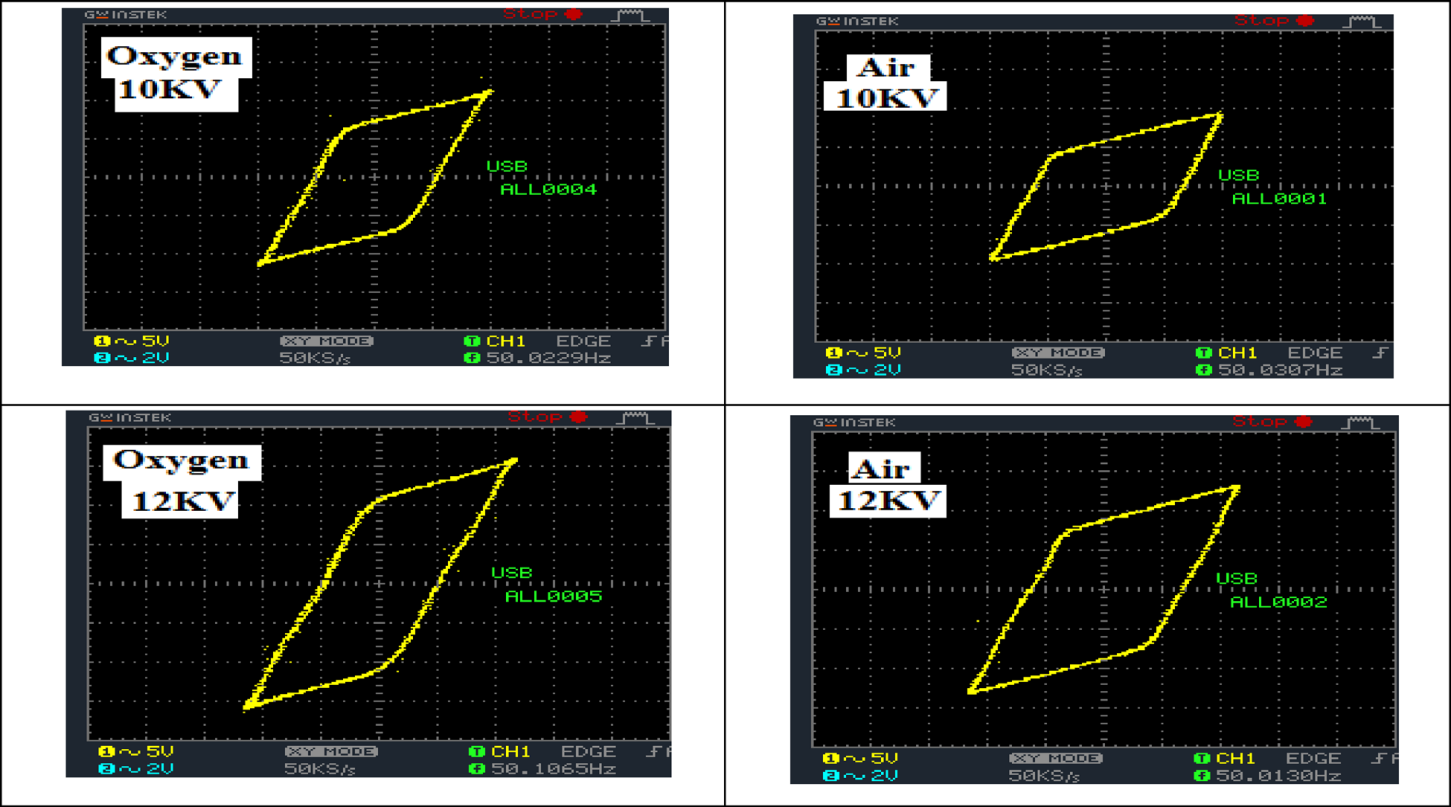
Lissajous figures of DBD for oxygen and air plasma at peak voltages of 10KV and 12 KV.

The experimental determination of the discharge DBD power has proven to be difficult, as the discharge power is used in a high number of short-lived micro-discharges. These difficulties can be avoided if the power is recorded from the voltage-charge Lissajous diagram^22,23^. Figure 4 displays Lissajous figures of DBD for oxygen and air plasma at peak voltages of 10KV and 12 KV.

The parallelogram area was monitored to increase with the raising of applied voltage, as the consumed power is relational to the parallelogram area. The consumed power has been estimated by multiplying the frequency of the AC power supply (50 Hz) by the parallelogram area. Table 1 shows the relation between the consumed power in the DBD cell and the applied voltage. It is noticed that the consumed power is very low (less than 30 W) regardless of the peak applied (even at the high value 12 KV). These results can be referred to the filamentary discharge performance, where the filament time is very short (a few tens of nanoseconds).

The table elegantly showcases how consumed power (P in watts) fluctuates with peak voltage (V in kilovolts). These power values are intricately derived from the area of the Lissajous parallelogram, a fascinating manifestation of voltage-current behavior in conjunction with frequency. We explore two distinct peak voltage levels: 10 kV and 12 kV. For each voltage, the resulting consumed power is presented for both air and oxygen plasma discharges. Thus, the values of 10 and 12 under V (kV) signify the peak voltage levels applied to the DBD cell, profoundly influencing the energy delivered and, consequently, the power consumed by the plasma.

**Table 1:** Relation between consumed power and peak voltage in a DBD cell for oxygen and air plasma.

Air DBD plasma		
V (kV)	10	12
P (W) = VI	10.15	15.9
**Oxygen DBD plasma**		
V (kV)	10	12
P (W) = VI	12.2	17.35

### Rheological measurements

Fenugreek galactomannan gums were modified using both oxygen and air plasma, and the rheological properties of the resultant viscous solutions were explored.

#### Effect of O_2_ plasma treatment

##### *Discharge power of 17.35 W*.

**Fig. 5:**
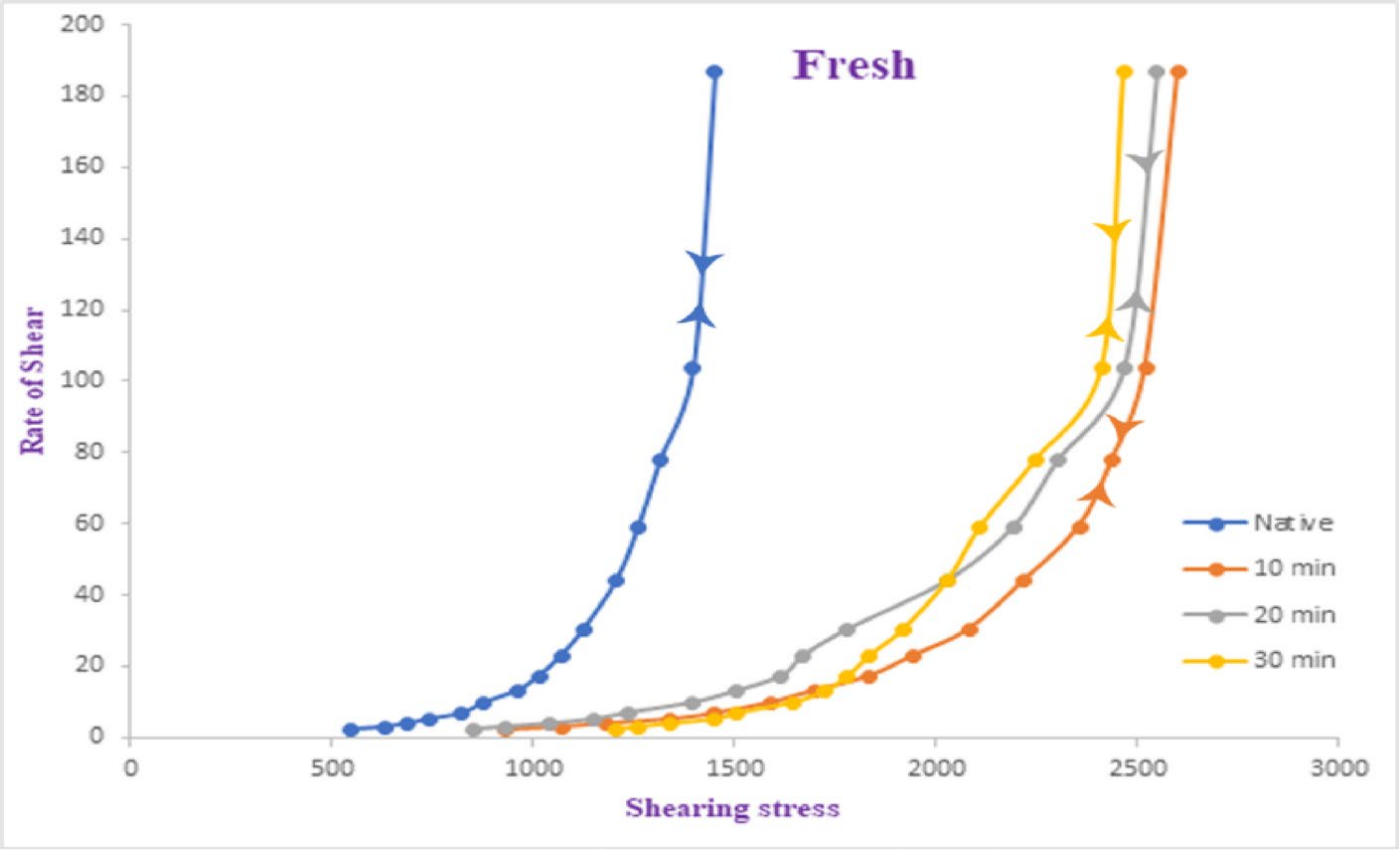
Rheological performance of both fresh and oxygen plasma-treated fenugreek gums at plasma exposure periods of 10, 20, and 30 min. The correlation level is identified as high (*p* ≤ 0.001).

Extracted galactomannan gum (2.5% solid content) was subjected to oxygen plasma treatment at a discharge power of 17.35 W for varying exposure times: 10, 20, and 30 min. The samples were dissolved in distilled water, and their rheological behavior was evaluated. Viscosity measurements were taken at different shear rates (s⁻¹) for both untreated (native) and treated samples. As illustrated in Fig. 5, the viscosity-shear rate relationship is nonlinear, confirming the non-Newtonian pseudoplastic nature of the gum. The similarity of the upward and downward flow curves further confirms that the gum exhibits reversible shear-thinning behavior. When subjected to high shear forces, the internal structure collapses, reducing viscosity. Upon release of the shear stress, the gum rapidly regains its original viscosity, indicating strong structural resilience. All plasma-treated samples demonstrated significantly higher viscosities compared to the native gum at all shear rates. For example, at a shear rate of 2.18 s⁻¹, the apparent viscosity increased from 547.60 Poise (native) to 930.92 Poise (10 min), 1204.72 Poise (20 min), and 848.78 Poise (30 min). This enhancement is more pronounced at lower shear rates, highlighting enhanced shear-thinning behavior. The sample treated for 20 min consistently exhibited the highest viscosity across most shear rates. At 13.12 s⁻¹, the viscosity reached 1724.94 Poise, compared to 1505.90 Poise for the 30-minute treated sample. Interestingly, the 30-minute treatment resulted in lower viscosities than the 20-minute treatment in most cases, indicating possible overexposure effects, such as polymer degradation or excessive oxidation. These results suggest the existence of an optimal plasma treatment duration, beyond which the structural integrity of the gum is compromised. Despite these differences, all samples retained the characteristic shear-thinning behavior, with viscosity decreasing as shear rate increased. This is typical of pseudoplastic materials and critical for applications where flow behavior under stress is important. The increase in apparent viscosity can be attributed to structural and chemical modifications induced by the plasma treatment. Plasma exposure likely introduces oxygen-containing functional groups, which enhance hydrophilicity and intermolecular interactions and potentially induce crosslinking. It has been reported that plasma-induced polymerization and crosslinking reactions contribute to viscosity increases, especially at lower shear rates^33^. Additionally, cold plasma treatment may promote the formation of hydrophilic linkages and stronger intermolecular bonds, enhancing the gel structure^34^. Overall, oxygen plasma treatment proves to be an effective green technology for modifying the rheological properties of galactomannan gum. The optimized 20-minute treatment significantly enhances the thickening capability, making it promising for food, pharmaceutical, cosmetic, and textile printing applications.

#### *Effect of O₂ Plasma Treatment at a Discharge Power of 12.20 W*

**Fig. 6:**
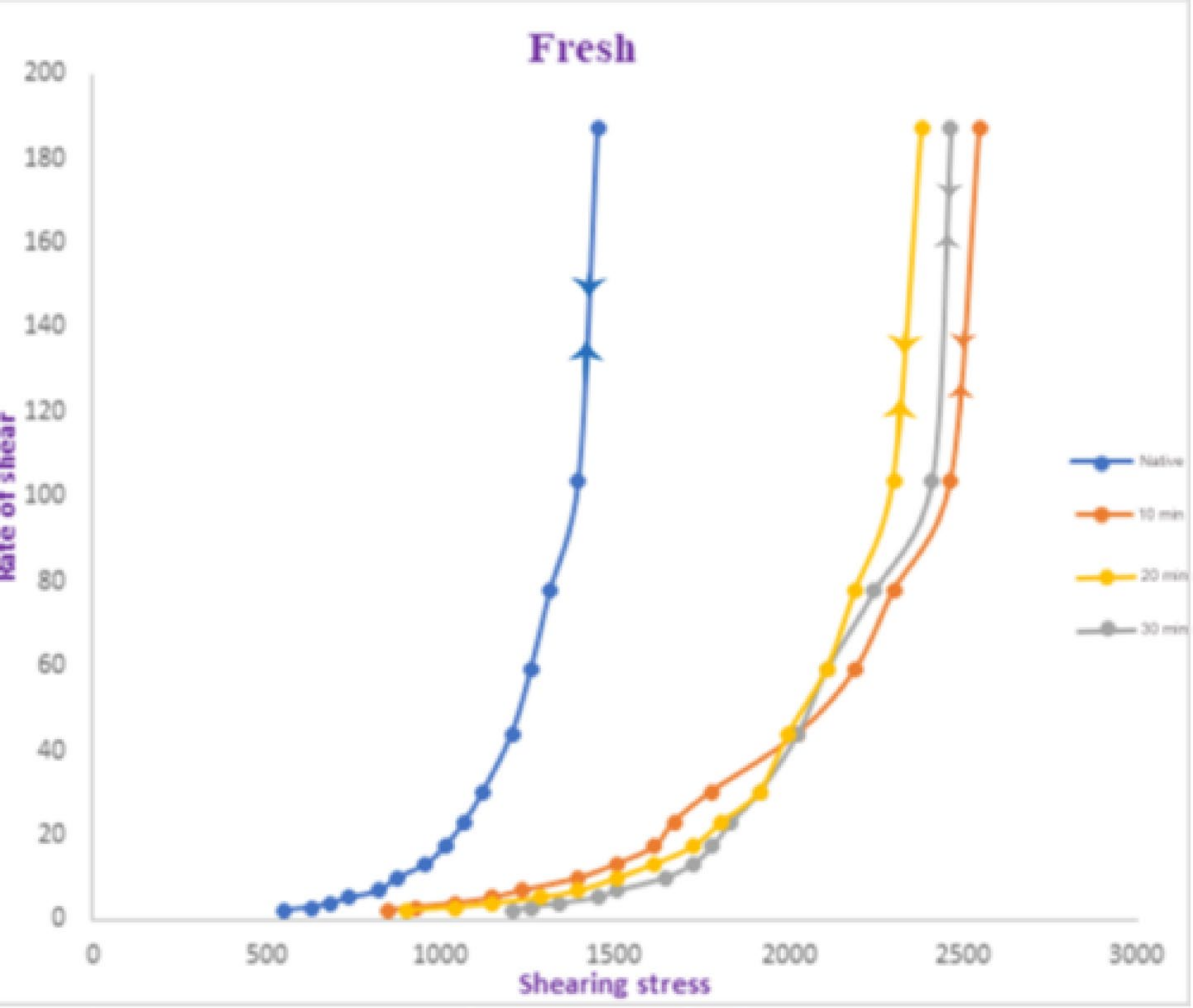
Rheological features of native, fresh, and oxygen plasma-treated fenugreek gum at exposure periods of 10, 20, and 30 min. The correlation level is identified as high (*p* ≤ 0.001).

The effect of oxygen plasma treatment at a discharge power of 12.20 W on the rheological properties of galactomannan gum was evaluated, with treatment durations of 10, 20, and 30 min. The results are illustrated in Fig. 6, focusing on the relationship between shear rate and shear stress and the corresponding apparent viscosities for native and treated samples. Similar to the behavior observed at the higher discharge power of 17.35 W, all samples—both treated and untreated—exhibited nonlinear flow curves, with the upward and downward sweeps coinciding, confirming the non-Newtonian pseudoplastic (shear-thinning) nature of galactomannan gum. As shear rate increases, viscosity decreases, and the material rapidly recovers its structure once the applied force is removed, indicating a robust and reversible structural response. Figure 6 reveals that plasma treatment at 12.20 W led to a consistent increase in apparent viscosity across nearly all tested shear rates, supporting the earlier findings at 17.35 W. For instance, at a shear rate of 2.18 s⁻¹, viscosity increased from 547.60 Poise (native) to 848.78, 903.54, and 1204.72 Poise after 10, 20, and 30 min of treatment, respectively. This trend held true at the highest shear rate of 187.10 s⁻¹, where viscosity rose from 1451.14 Poise (native) to 2546.34, 2382.06, and 2464.20 Poise, respectively. This increase in viscosity is attributed to plasma-induced chemical modifications, particularly the generation of functional groups that promote crosslinking within the galactomannan matrix. These modifications enhance intermolecular interactions, increase resistance to deformation, and improve the material’s ability to retain its structure under stress. Plasma exposure effectively creates more covalent linkages, which stabilize the gum network and increase its apparent viscosity. Compared to the higher discharge power, the optimum exposure time for achieving maximum viscosity appears to shift at 12.20 W. At low shear rates, viscosity steadily increases with longer treatment durations, with the 30-minute treatment consistently yielding the highest values. For example, at 13.12 s⁻¹, the viscosity rises from 1505.90 (10 min) to 1615.42 (20 min) and 1724.94 Poise (30 min). However, at higher shear rates, the difference between the 20- and 30-minute treatments becomes less pronounced, and in some cases, the 20-minute sample may even perform slightly better. This suggests that longer exposure times may be more beneficial at lower discharge powers, potentially due to slower or less intense chemical activation at reduced energy input. When it is comparing the effects of the two discharge powers. Higher discharge power (17.35 W) results in greater viscosity increases at shorter exposure times (10–20 min). But at lower power (12.20 W) requires longer exposure (up to 30 min) to achieve similar or slightly improved viscosity enhancement, particularly at low shear rates. For instance, at 2.18 s⁻¹, the 20-minute treatment at 17.35 W raised viscosity to 1204.72 Poise, while the same treatment at 12.20 W achieved 903.54 Poise. This indicates a less intense modification effect at the lower power setting, requiring extended exposure for optimal outcomes. Across all plasma-treated samples, the shear-thinning behavior of galactomannan gum remains intact, a crucial feature for its application in flow-sensitive products. The reversible collapse and rebuild of structure under and after shear stress highlight the material’s ability to perform effectively in dynamic processing environments.

Oxygen plasma treatment at a discharge power of 12.20 W effectively enhances the apparent viscosity of galactomannan gum, particularly with longer exposure times. The optimum treatment duration appears more variable compared to higher power settings, with 30 min often producing the highest viscosities, especially at low shear rates. These findings underscore the need to optimize plasma parameters—including power and duration—based on specific application needs. Furthermore, the differences in structural response between the two power levels suggest that discharge power influences the rate and extent of molecular modification, affecting how intermolecular interactions develop over time. Additional investigations into the chemical and morphological changes induced by plasma at varying power levels would help refine this promising green modification technique for tailored rheological control in industries such as food, pharmaceuticals, cosmetics, and textile printing.

#### ***Effect of air plasma treatment at discharge power of 15.90 W***

**Fig. 7:**
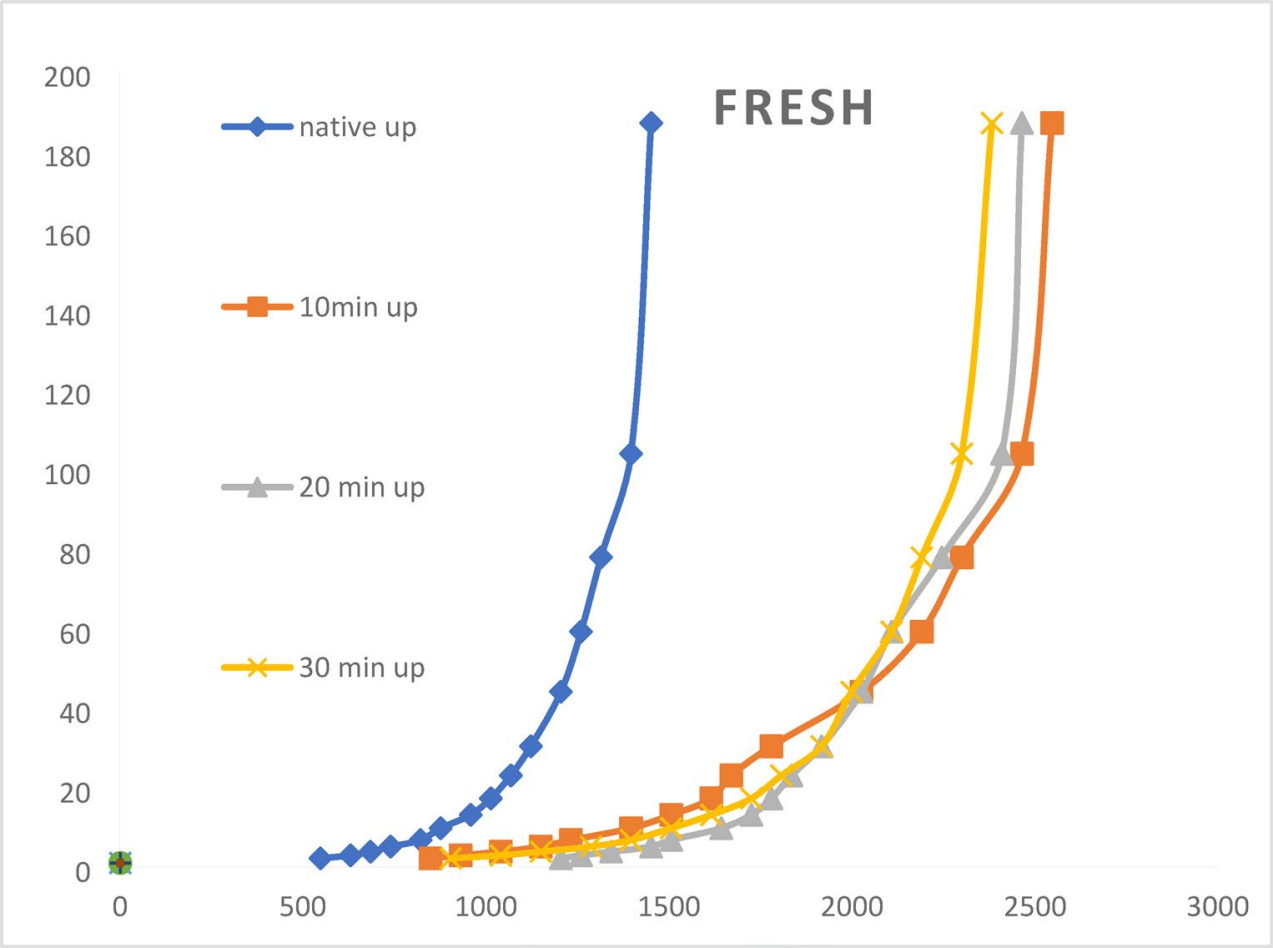
Rheological features of native, fresh, air plasma-treated fenugreek gums at different exposure periods of 10, 20, and 30 min. The correlation level is identified as high (*p* ≤ 0.001).

the impact of air plasma treatment at a discharge power of **15.90 W** on the rheological characteristics of the galactomannan gum thickener. Figure 7 illustrates the relationship between shear rate and shearing stress for the native galactomannan gum and samples treated with air plasma for 10, 20, and 30 min at a discharge power of 15.90 W. Consistent with the oxygen plasma treatments discussed earlier, the curves demonstrate a non-linear relationship with coincident upward and downward paths, confirming the pseudoplastic (shear-thinning) behavior of the gum solutions. This indicates that the viscosity decreases under increasing shear stress and recovers upon its removal. Figure 7 provides the apparent viscosity (in Poise) of the native and air plasma-treated galactomannan gum at various shear rates. Similar to the oxygen plasma treatments, exposure to air plasma at 15.90 W leads to an increase in the apparent viscosity of the galactomannan gum across all tested shear rates compared to the native sample. This confirms that air plasma is also an effective method for modifying the thickening properties of the gum. A notable trend with air plasma treatment at this power is the direct correlation between the plasma exposure duration and the apparent viscosity. As the treatment time increases from 10 to 20 to 30 min, the apparent viscosity generally increases at each measured shear rate. This suggests that longer exposure to air plasma at 15.90 W promotes further structural modifications within the gum, leading to enhanced thickening ability. The text suggests that the increased viscosity can be attributed to the creation of more bonds within the macromolecular structure of the galactose and mannose units of the gum. The introduction of more functional groups onto the gum surface by the air plasma likely facilitates increased intermolecular interactions and bond formation between the glucose units in the galactomannan structure, particularly at the ends of the glucose chains. This enhanced network formation results in higher viscosity. Despite the increase in viscosity, all air plasma-treated samples continue to exhibit pseudoplastic behavior, as their apparent viscosity decreases with increasing shear rate. The air plasma treatment seems to enhance the magnitude of the viscosity without altering the fundamental shear-thinning characteristic of the galactomannan gum. At similar treatment times, the magnitude of viscosity enhancement achieved with air plasma at 15.90 W appears to be comparable to, and in some cases even slightly higher than, that achieved with oxygen plasma at 12.20 W (referring back to the previous discussion).

Unlike the oxygen plasma treatment at 17.35 W, where an optimal treatment time of 20 min was observed, the air plasma treatment at 15.90 W shows a consistent increase in viscosity up to the longest tested duration of 30 min. This suggests that the type of plasma gas and the discharge power significantly influence the optimal treatment parameters. In conclusion, air plasma treatment at a discharge power of 15.90 W effectively enhances the apparent viscosity of galactomannan gum, and this enhancement is directly proportional to the plasma exposure time within the tested range (10–30 min). This increase is likely due to the introduction of functional groups and the formation of additional bonds within the gum’s structure, leading to stronger intermolecular interactions. The treated gum retains its desirable shear-thinning behavior, making air plasma a promising technique for tailoring the rheological properties of galactomannan gum for various applications.

#### Discharge power of 10.15 W

**Fig. 8:**
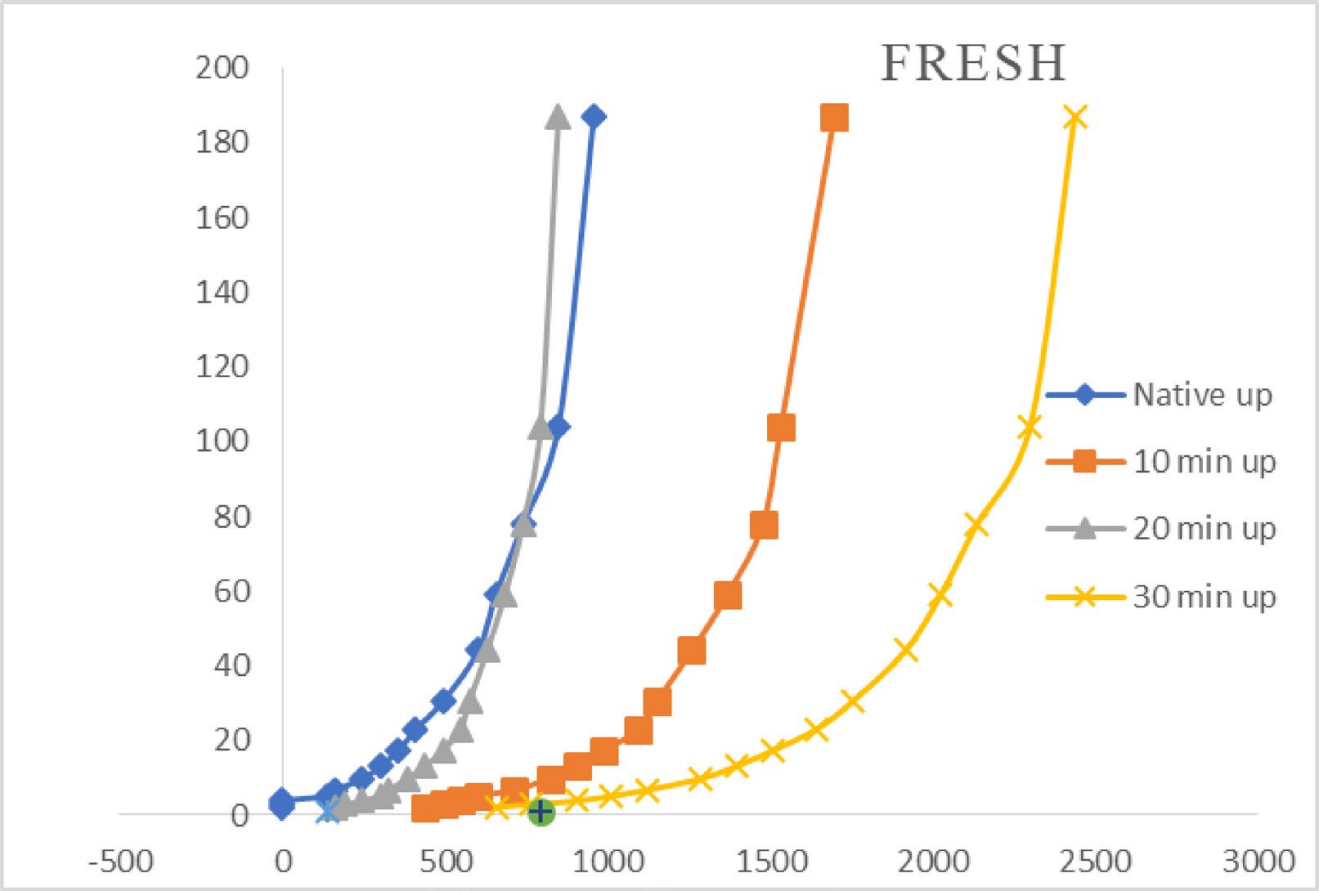
Rheological measurements of native, fresh, and air plasma-treated fenugreek gums at different exposure periods of 10, 20, and 30 min. The correlation level is identified as high (*p* ≤ 0.001).

The effect of air plasma treatment at a discharge power of 10.15 W on the galactomannan gum thickener, as depicted in Fig. 8, reveals some interesting trends. Figure 8 displays the relationship between shear rate and shearing stress for the native gum and samples treated with air plasma for 10, 20, and 30 min at 10.15 W. The curves, like those observed at higher discharge powers, exhibit a non-linear relationship with coincident up and down sweeps, confirming the pseudoplastic (shear-thinning) nature of the gum solutions. The text reiterates that under high shear forces, a temporary structural collapse occurs, leading to a decrease in viscosity. Consistent with the trend observed at 15.90 W, the apparent viscosity tends to increase with increasing air plasma exposure time from 10 to 30 min at this lower power. This suggests that longer treatment durations at 10.15 W continue to promote structural changes within the gum that enhance its viscosity. Comparing the viscosity values at (10.15 W) and (15.90 W) for the same treatment times, it appears that the magnitude of viscosity enhancement is generally lower at the 10.15 W discharge power for most shear rates. For example, at a shear rate of 2.18 s⁻¹ and a 30-minute treatment, the viscosity is 1204.72 Poise at 10.15 W, which is the same as at 15.90 W. However, at other shear rates, the values at 15.90 W tend to be higher. This suggests that a higher discharge power leads to a more pronounced increase in the apparent viscosity of the galactomannan gum for a given treatment time. The energy input from the plasma likely plays a significant role in the extent of structural modification. As with the other treatment conditions, the air plasma-treated samples at 10.15 W continue to exhibit pseudoplastic behavior, with viscosity decreasing as the shear rate increases. The plasma treatment enhances the overall viscosity level without altering this fundamental rheological characteristic.

### Scanning electron microscopy

**Fig. 9:**
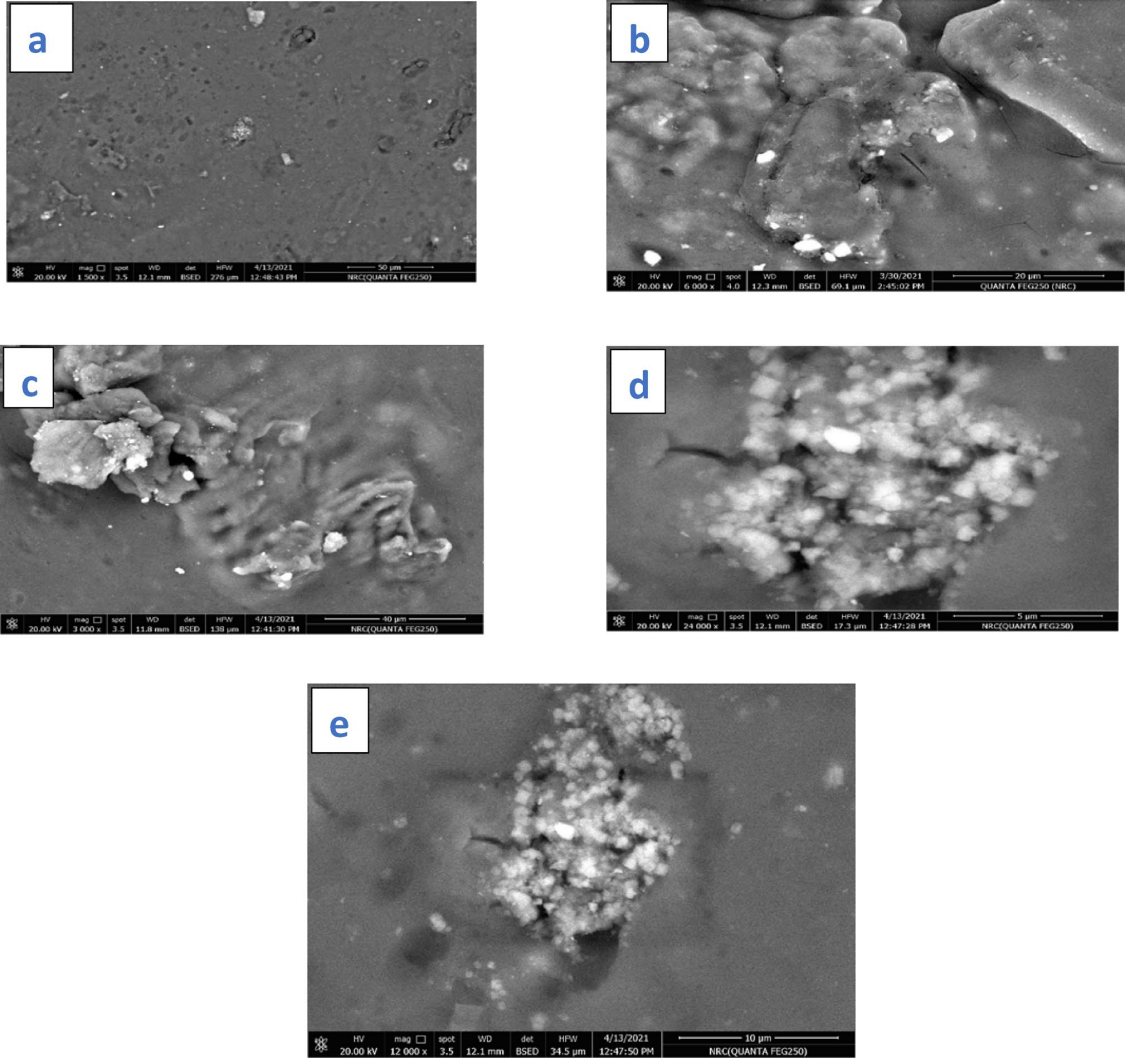
SEM images of untreated fenugreek film, air plasma-treated fenugreek at exposure period of 30 min and discharge powers of 10.15 W and 15.9 W, and oxygen plasma-treated fenugreek at exposure periods of 30 min and discharge power of 12.20 W (a-e), demonstrating similar morphologies.

The structure morphology of the fenugreek film was explored by SEM, FTIR, and EDX spectroscopy. SEM images of the control and DBD-cured fenugreek films are depicted in Fig. 9. The untreated fenugreek has shown a compact surface, as shown in Fig. 9a. The air and oxygen plasma-treated fenugreek at different discharge powers and different exposure periods show porous structures, as shown in Fig. 9b-e. The treated samples revealed laminated structures and high surface roughness due to the plasma-driven surface etching of the gum surface. The cold plasma can modify the hydrocolloid microstructure via chemical (e.g., degradation and chain breakage) and/or physical (e.g., separating some units in the gum structure) mechanisms^35^. Plasma etching can improve the surface energy of the polysaccharide galactomannan, improving its hydrophilicity toward a better extraction process of the water-soluble fenugreek^36^. The high density of the neutral active species in oxygen plasma results in better etching of the fiber surface, forming grooves and cracks^22–27^. The oxygen-based free radicals improve the surface polarity by presenting new polar hydroxyl and carboxyl derivatives either instantly after plasma treatment or during the treatment process^37^. The changes in the fenugreek morphology owing to oxygen plasma curing at 17.35 W for 30 min are more efficient than the air plasma curing, as shown in Fig. 9b-e. Oxygen plasma generates cracks as well as introducing a hydrophilic protein moiety and numbers of hydrogen bonds^38^. The swelling of the treated samples, either by air or oxygen plasma, increased as compared to the untreated sample, which could be connected to depolymerization of polysaccharides stimulated by the plasma-induced active species. This results in the creation of smaller fragments with a better water affinity^39^.

### Energy-dispersive X-ray

**Fig. 10:**
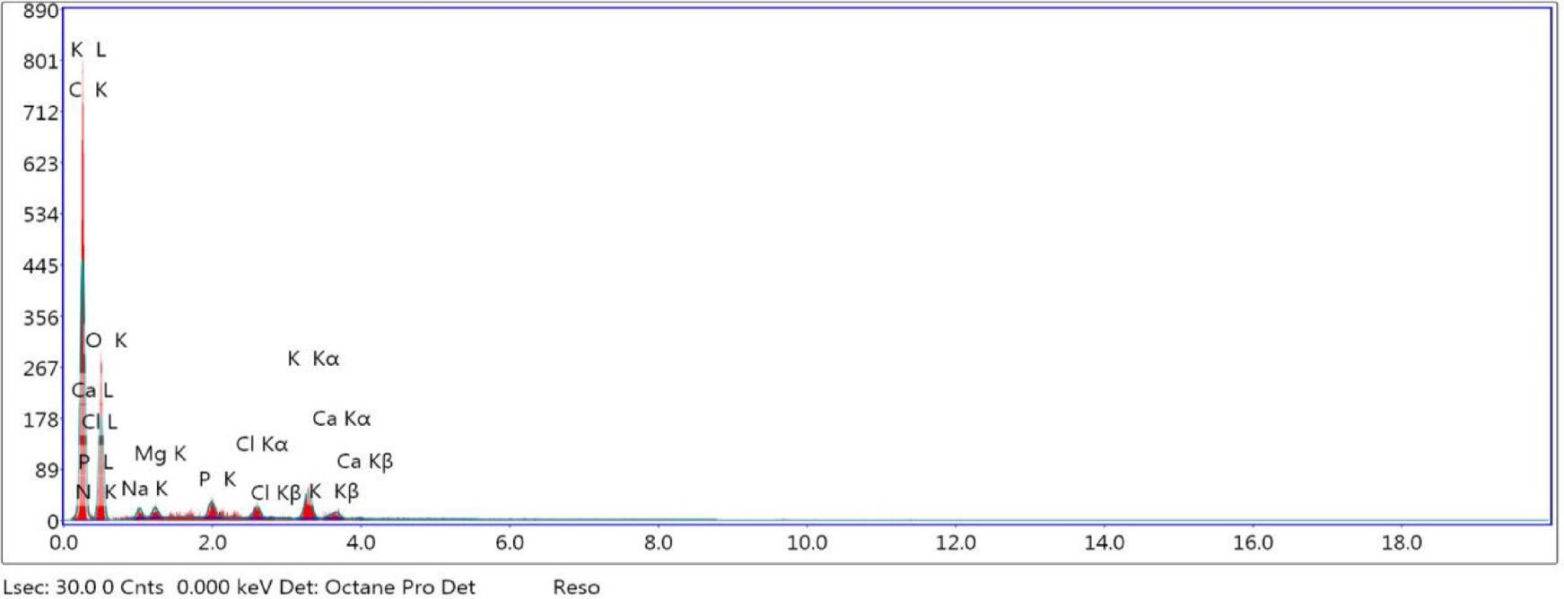
EDX diagram of untreated fenugreek film.

**Fig. 11:**
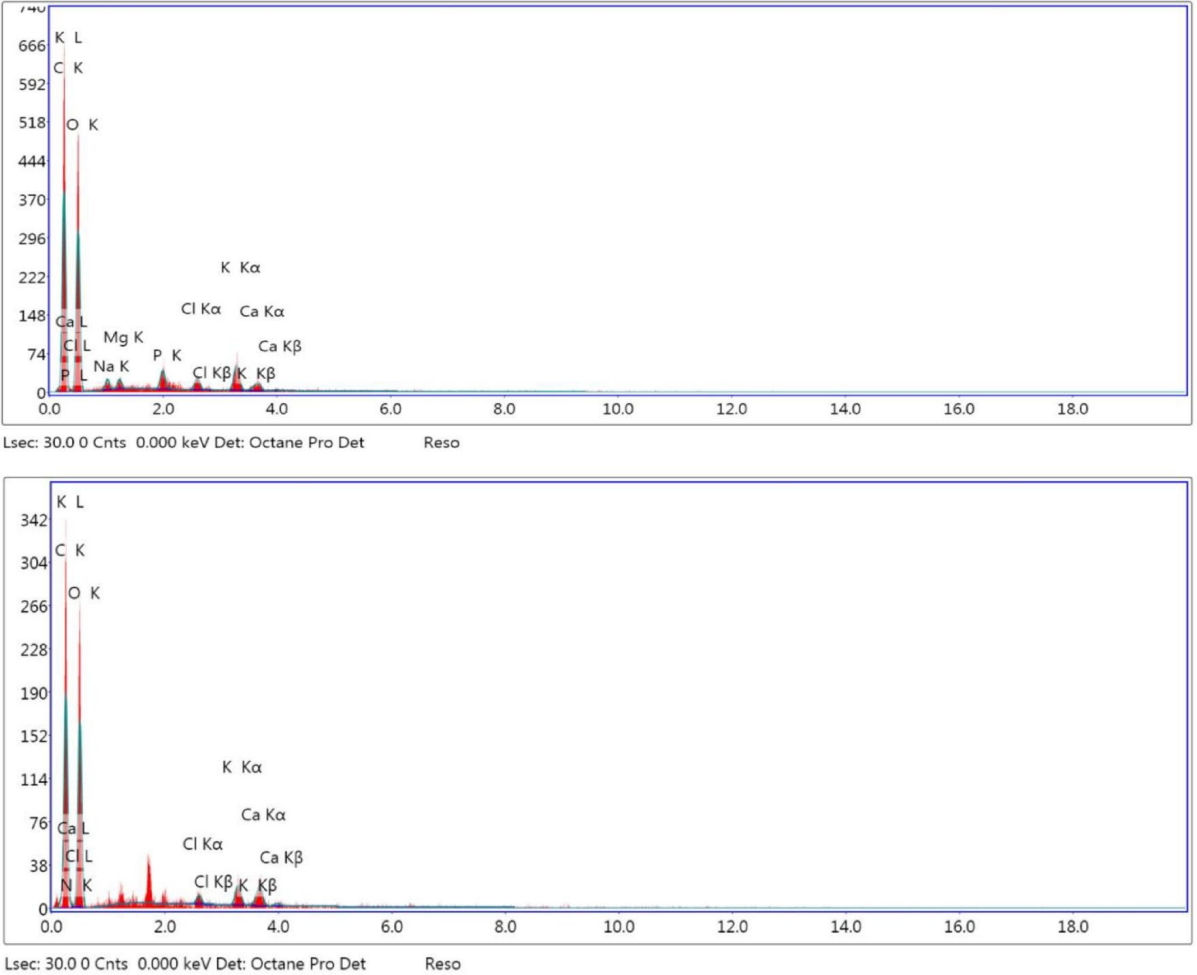
EDX diagram of air plasma-cured film at discharge powers of 10.5 W (*top*) and 15.9 W (*bottom*) and an exposure period of 30 min.

**Fig. 12:**
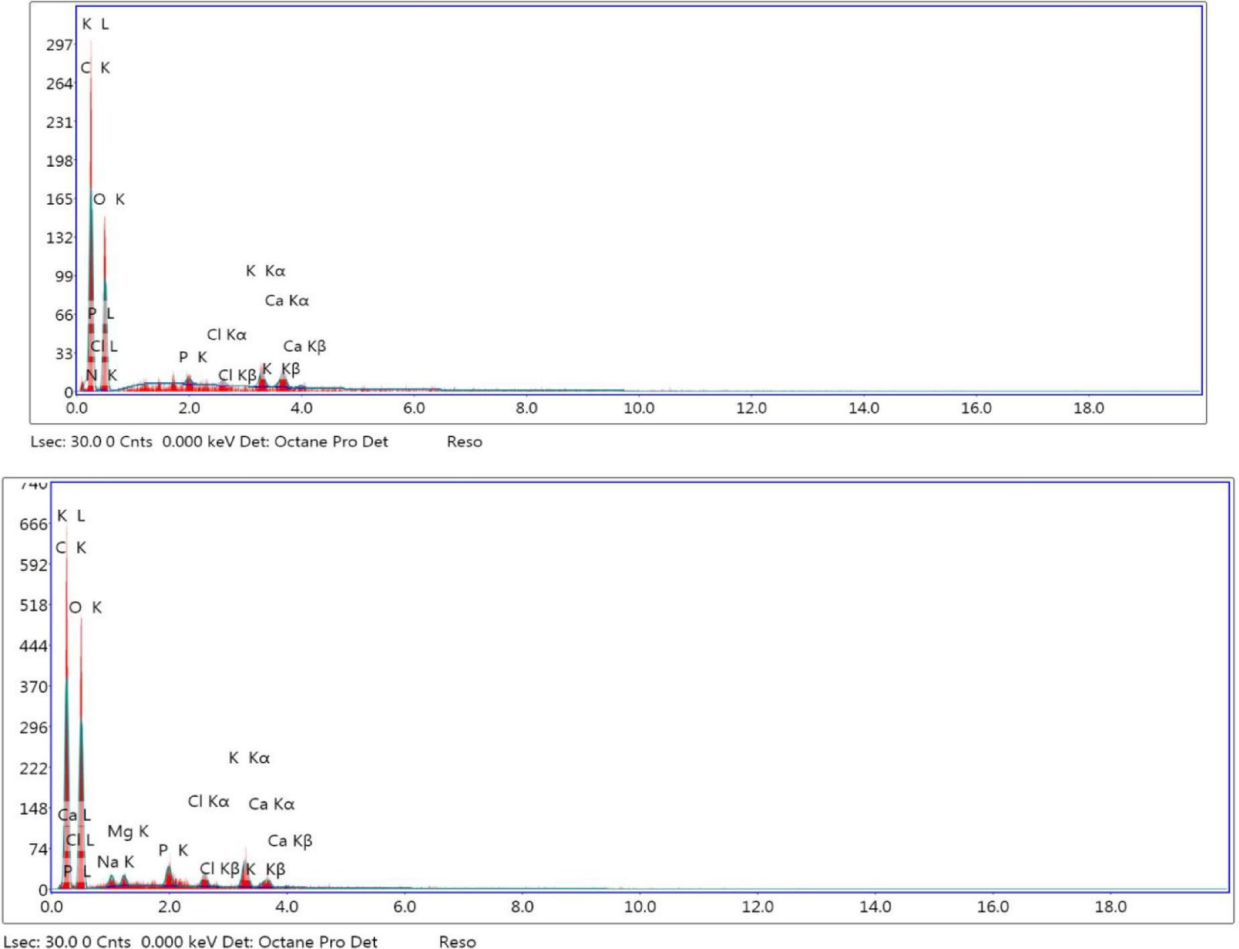
EDX diagram of oxygen plasma-cured film at discharge powers of 12.2 W (*top*) and 17.35 W (*bottom*) and an exposure period of 30 min.

Fenugreek seeds have been known as a good source of minerals like potassium, iron, phosphorus, magnesium, manganese, copper, and calcium. The elemental compositions of the plasma-activated fenugreek films were studied by using EDX spectroscopy, as shown in Table 2. The EDX diagram of untreated fenugreek film is shown in Fig. 10. EDX diagrams for air and oxygen plasma-cured films at discharge powers of 100 W and 120 W and an exposure period of 30 min are shown in Figs. 11 and 12, respectively. EDX was examined at three different sites, demonstrating identical compositions. Oxygen (O), calcium (Ca), potassium (K), nitrogen (N), carbon (C), and phosphorus (P) are the elements that were detected by EDX. The prepared smart films consisted mainly of carbon, oxygen, and nitrogen.

**Table 2:** EDX analysis for untreated and plasma-treated films at three scanned sites (P_1_, P_2_, and P_3_).

Plasma condition	Elemental content (wt%)					
	C	O	*N*	*P*	Ca	K
*Control*						
P_1_	44.97	48.98	1.71	0.67	1.25	2.91
P_2_	44.86	48.72	1.83	0.88	1.02	2.72
P_3_	44.92	48.88	1.85	0.75	1.13	2.90
*Air plasma at 10.5 W *,*30 min*						
P_1_	38.61	42.03	1.55	0.77	1.45	2.19
P_2_	39.67	42.73	1.74	0.86	1.42	2.94
P_3_	39.73	42.03	1.65	0.75	1.23	2.89
*Air plasma at 15.9 W*,* 30 min*						
P_1_	42.5	50.64	2.84	0.58	1.88	1.59
P_2_	51.44	45.54	1.36	0.42	1.8	1.43
P_3_	47.43	43.16	2.09	0.46	1.85	1.56
*O*_*2*_ *plasma at 12.2 W *,*30 min*						
P_1_	46.39	48.32	1.89	0.47	1.74	2.18
P_2_	46.28	49.65	1.90	0.51	1.23	2.41
P_3_	46.25	50.01	1.97	0.54	1.64	2.04
*O*_2_ *plasma at 17.35 W, 30 min*						
P_1_	44.66	52.06	1.43	0.52	1.94	2.88
P_2_	44.64	50.13	1.53	0.82	1.86	1.99
P_3_	45.31	50.21	1.49	0.84	1.97	2.21

### Transmission electron microscope

**Fig. 13:**
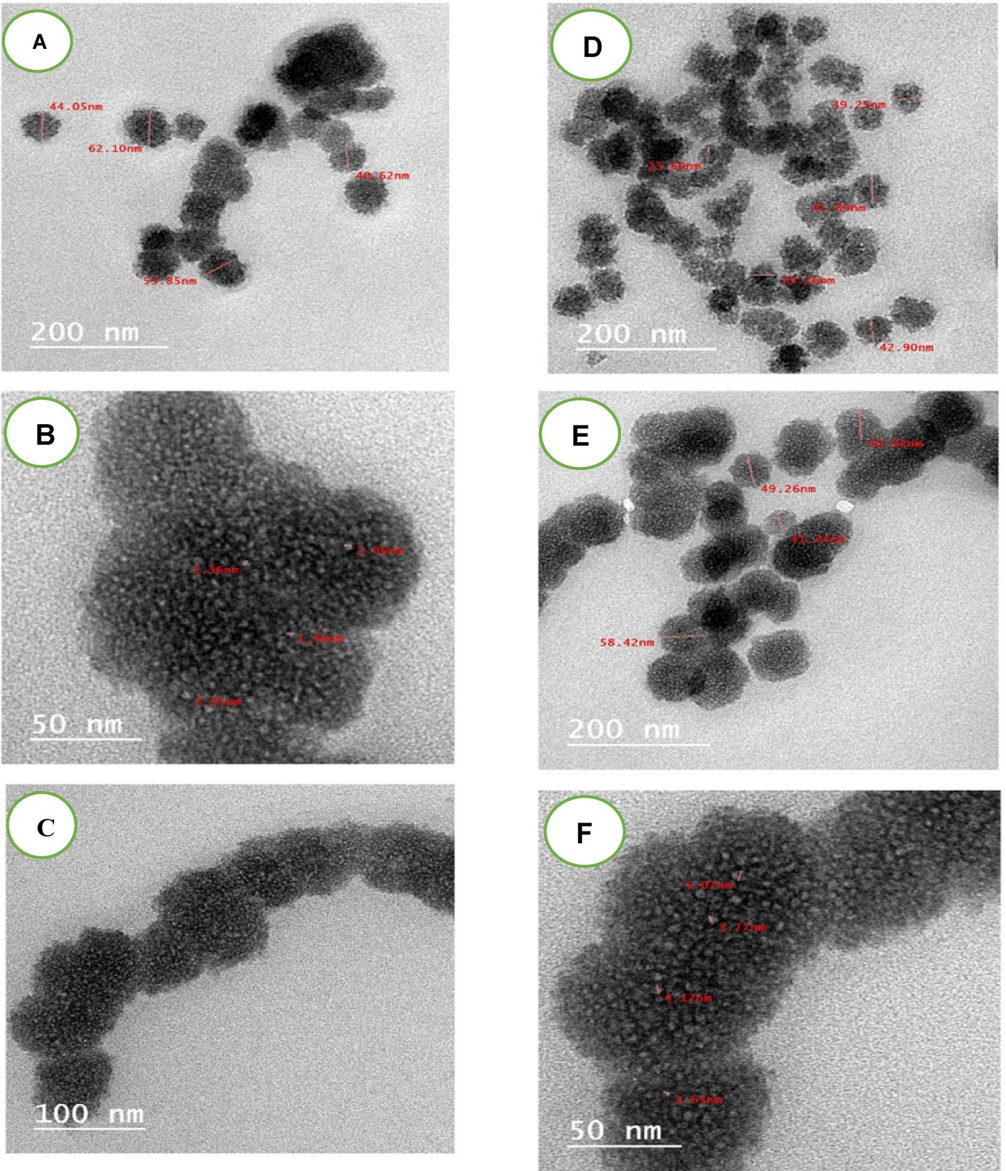
TEM images of fenugreek film after exposure to oxygen plasma at 10.12 W and 17.35 W (A-F) for a period of 30 min.

Figure 13a–f presents the TEM images of fenugreek films treated with air and oxygen plasma at a power of 10.12 W for an exposure time of 30 min. A comparison of images in Fig. 13a–f, which show the effects of both plasma treatments, reveals that the fenugreek particles were broken down, leading to an increased surface area as a result of surface interaction and plasma etching. Following this fragmentation, the particles coagulated to form capsule-like structures, which appear smaller in the oxygen plasma-treated samples compared to those treated with air plasma. The particle diameters observed in the air plasma-treated samples were 44.05, 59.85, and 62.10 nm, while those in the oxygen plasma-treated samples measured 25.60, 39.25, and 42.90 nm, indicating a more pronounced size reduction with oxygen plasma treatment.

### Fourier-transform infrared spectrophotometer

**Fig. 14:**
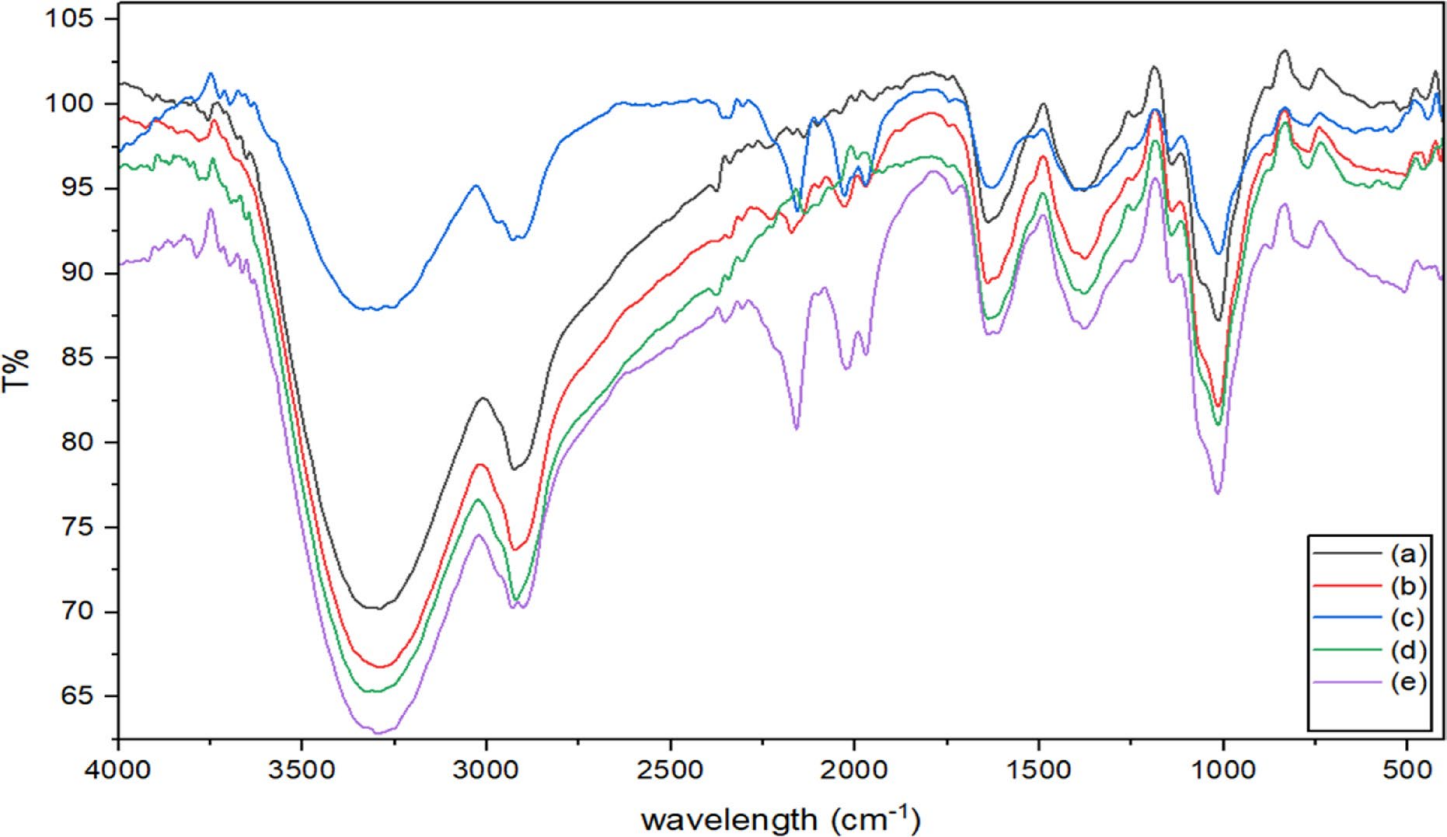
FTIR the untreated and plasma-treated thickeners (**a**) untreated film, (**b**) plasma-treated film with air plasma at power 10.5 watts, (**c**) plasma-treated film with oxygen plasma at power 12.2 watts, (**d**) plasma-treated film with air plasma at power15.9 watts, (**e**) plasma-treated film at power 17.35 watts.

The characteristic peaks of the untreated and plasma-treated thickeners are shown in aFig. 14. The infrared spectrum of the untreated fenugreek film is displayed in Fig. 14. The band at 3365 cm^–1^ is assigned to the N–H stretching vibration (amide A of protein), and the O–H stretching vibration of the starch fiber. The shoulder peak detected at 3130 cm^–1^ could be assigned to the secondary amide. This could be attributed to Fermi resonance from the N–H peak (stretching) with the amide II peak overtone in trans-amide or from the N–H peak (stretching) combined with the stretching carbonyl and N–H in-plane binding in cis-amide^39^. The peaks monitored at 2961, 2928, and 2846 cm^–1^, are attributed to symmetrical and asymmetrical stretching vibrations of the C–H groups. The relatively medium peak at 1743 cm^–1^ is attributed to the carbonyl stretching vibration of lipids^40^. The amide groups are distinguished by N–H deformation, and stretching vibrations of N–H and C = O^41^. The amide I peak is attributed to the carbonyl stretching. In the case of a primary amide, the amide II peak is ascribed to the NH_2_ deformation. In the case of secondary amides, the amide II peak is assigned to mixed vibrations of C–H stretching and N–H bending. Strong peaks were detected at 1241 cm^–1^ (secondary amide III, N–H bending), 1541 cm^–1^ (amide II, N–H bending), and 1658 cm^–1^ (amide I, C = O), proving a solid-state protein^42^. The peak detected at 1460 cm^–1^ is ascribed to the C–H bending deformation, whereas the peak monitored at 1401 cm^–1^ is attributed to the bending vibration of the COO^–^ substituents in primary amides. Aromatic alcohols caused the appearance of a peak at 1241 cm^–1^ owing to the N–H bending. The absorption band at 1161 cm^–1^ (C–O) is assigned to starch. The band detected at 869 cm^−1^ is ascribed to the anomeric configuration and glycosidic bonds (β-conformation) in galactomannan^43^. Upon germination, fenugreek is composed essentially of ash, fibers, lipids, protein, and soluble sugar, as well as low contents of fats and starch^44^. The infrared spectra of the air/oxygen plasma-cured samples are shown in Fig. 14. The plasma curing process was performed over various intervals and at different levels of discharge power. Plasma curing has been an effective technique in the modification of thickeners by enhancing swelling properties and viscosity. This is caused by the formation of polar substituents, such as − C = O, −CO−, −COOH^36^. The sharp peaks monitored at 3419 cm^–1^, 2927 cm^–1^, 1680–1745 cm^–1^, and 2900 cm^–1^, are ascribed to hydroxyl (O − H), amid (C = N), carbonyl (C = O) and C-H bonds, respectively. Air/oxygen plasma curing results in oxidation of polysaccharides chains. The glucoside hydroxyl is oxidized to carboxyl and carbonyl groups, which disrupts the hydrogen-bonding in the thickener, promoting better swelling of the treated fenugreek in aqueous media. The air plasma-induced oxidized components are slightly lower than that of oxygen plasma. This could be attributed to the higher density of free radicals and fractures of glucoside bonds to produce active carbonyl groups on the surface of treated fenugreek films Fig. 14.

### Antibacterial activity

The antimicrobial potential of both untreated and plasma-treated fenugreek films was explored through the innovative disc agar diffusion method. This investigation focused on a range of pathogens, including E. coli (ATCC 25933; a notorious Gram-negative bacterium), S. aureus (ATCC 6538-P; a resilient Gram-positive bacterium), Aspergillus niger (NRRL-A326; a formidable fungus), and C. albicans (ATCC 10231; a common yeast). The results promise to shed light on the efficacy of these films in combating microbial threats. Potato Dextrose Agar (PDA) seeded with 0.1 mL (10^6^ cells/mL) of the fungus inoculum was utilized to assess the antifungal activity. In the case of yeast and bacteria, the nutrient agars were inoculated regularly with 0.1 ml of 10^5^−10^6^ cells/mL. The nutrient agars were maintained at 4 °C for 2–4 h to allow maximum diffusion. The nutrient agars were incubated at 37 °C for 24 h for bacterial pathogens and at 30 °C for 48 h in the upright location to allow maximum growth of organisms. The antimicrobial performance was recorded by recording the diameter of the inhibition zone (mm) as demonstrated in Table 3. The plasma treatment has been discovered to significantly enhance the antibacterial properties of fenugreek against both Gram-negative and Gram-positive bacterial pathogens, irrespective of the specific plasma curing conditions employed. Notably, O_2_ plasma curing stands out as notably more effective than air plasma treatment. This remarkable improvement can be attributed to an increase in active oxygen species, such as hydroxyl radicals, alongside the etching process that occurs in the fenugreek film. The active nitrogen and oxygen species produced during the plasma discharge engage with liquids, resulting in the generation of a diverse array of reactive species, including nitrite, H^+^, H_2_O_2_, ozone, nitrate, OH, singlet O_2_, and superoxide^45^. However, the quest to identify these species and to accurately measure their concentrations in the liquid phase remains a challenge. This complexity arises from the multitude of simultaneous chemical interactions and energy exchanges, creating a tapestry of intricate kinetics that yield a fascinating variety of reactive species^46,47,48^. Furthermore, atmospheric cold plasma treatment has been shown to elevate the antibacterial activity of fenugreek gum, which is particularly susceptible to bacterial attack and may undergo degradation of its polymeric chains. This innovative treatment not only showcases the potential of fenugreek but also opens new avenues for enhancing natural antibacterial agents.

**Table 3:** Antibacterial activity of untreated and plasma-treated Fenugreek films using CFU.

Sample	Condition of plasma treatment		Staphylococcus aureus		Escherichia coli	
	Power (wt.)	Time treatment (min)	No. of colonies (10^−3^)	*R* (%)	No. of colonies (10^−3^)	*R* (%)
**Bacterial control**	-	-	43	-	25	-
**Native**	-	-	10	76.74	0	100
**Treatment Film with air plasm**	10.15	20 30	15 4	65.12 90.70	2 0	92.00 100.00
	15.9	20 30	6 5	78.9 88.37	10 13	46.00 48.00
**Treatment Film with O**_**2**_ **plasma**	12.2	20 30	7 2	83.72 95.35	8 6	68.00 76.00
	17.35	20 30	3 0	93.02 100.00	0 0	100.00 100.00

## Conclusions

The exploration of atmospheric cold plasma’s impact on fenugreek gum has revealed remarkable findings. Plasma treatment has significantly bolstered the membrane integrity of the extracted gum, enhancing its overall quality. At elevated discharge powers, we observed a striking increase in the apparent viscosity of galactomannan, signaling a dramatic improvement in its rheological behavior. Notably, this enhancement was achieved without compromising the gum’s inherent shear-thinning characteristics. Advanced analyses, including Scanning Electron Microscopy (SEM) and Energy Dispersive X-ray Spectroscopy (EDX), demonstrated that the samples treated with air or oxygen plasma experienced greater swelling compared to their untreated counterparts. This phenomenon can be attributed to the depolymerization of polysaccharides spurred by the reactive species produced during plasma exposure. Furthermore, Fourier Transform Infrared Spectroscopy (FTIR) analysis confirmed significant modifications in the gum’s functional groups, altering the delicate balance between polar and nonpolar substituents. These chemical transformations subsequently influenced both surface tension and antibacterial activity.

The plasma-modified samples showcased enhanced antibacterial properties, likely resulting from increased hydrophobicity. In addition, plasma treatment remarkably improved the gum’s ability to lower surface tension. While both treated and untreated samples exhibited shear-thinning behavior, the treated samples stood out, demonstrating higher viscosity and superior viscoelastic performance.

Atmospheric cold plasma—particularly through the innovative application of dielectric barrier discharge (DBD) plasma—emerges as an extraordinarily effective, non-thermal, and eco-friendly method for amplifying the rheological, structural, and functional attributes of fenugreek gum. This groundbreaking approach not only elevates extraction efficiency but also paves the way for the development of multifunctional bioactive films without reliance on chemical agents, ushering in a new era of sustainable and innovative applications.

## Data Availability

The data that support the findings of this study are available from the corresponding author upon reasonable request.
